# Gut microbiome and inflammation among athletes in wheelchair in a crossover randomized pilot trial of probiotic and prebiotic interventions

**DOI:** 10.1038/s41598-024-63163-z

**Published:** 2024-06-04

**Authors:** Ezra Valido, Simona Capossela, Marija Glisic, Anneke Hertig-Godeschalk, Alessandro Bertolo, Gerold Stucki, Joelle Leonie Flueck, Jivko Stoyanov

**Affiliations:** 1 Swiss Paraplegic Research, 6207 Nottwil, Switzerland; 2 Institute of Social and Preventive Medicine (ISPM), University of Bern, 3012 Bern, Switzerland; 3 Institute of Sports Medicine, Swiss Paraplegic Centre, 6207 Nottwil, Switzerland; 4 Department of Orthopedic Surgery, University of Bern, Bern Inselspital, 3012 Bern, Switzerland; 5 Faculty of Health Sciences and Medicine, University of Lucerne, 6003 Lucerne, Switzerland

**Keywords:** Inflammation, Inflammatory markers, Metabolism, Linear mixed-effect models, Microbiome, Probiotics, Prebiotics, Spinal cord injury, Microbiology, Gastroenterology, Medical research, Molecular medicine

## Abstract

Disorders related to gut health are a significant cause of morbidity among athletes in wheelchair. This pilot feasibility trial aims to investigate whether probiotics compared to prebiotics can improve inflammatory status and gut microbiome composition in elite athletes in wheelchair. We conducted a 12-week, randomized, cross-over controlled trial involving 14 elite Swiss athletes in wheelchair. Participants were given a multispecies-multistrain probiotic or prebiotic (oat bran) daily for 4 weeks (Clinical trials.gov NCT04659408 09/12/2020). This was followed by a 4-week washout and then crossed over. Thirty inflammatory markers were assessed using bead-based multiplex immunoassays (LegendPlex) from serum samples. The gut microbiome was characterized via 16S rRNA sequencing of stool DNA samples. Statistical analyses were conducted using linear mixed-effect models (LMM). At baseline, most athletes (10/14) exhibited low levels of inflammation which associated with higher gut microbiome alpha diversity indices compared to those with high inflammation levels. The use of probiotic had higher decrease in 25 (83%) inflammatory markers measured compared to prebiotic use. Probiotic has the potential in lowering inflammation status and improving the gut microbiome diversity. The future trial should focus on having sufficient sample sizes, population with higher inflammation status, longer intervention exposure and use of differential abundance analysis.

## Introduction

### Background

Individuals in wheelchair may have various underlying conditions, ranging from traumatic or non-traumatic injuries to the spinal cord or brain, degenerative diseases affecting the central nervous system (CNS) such as multiple sclerosis, or congenital conditions such as spina bifida. The involvement of the CNS, particularly the spinal cord, often leads to multi-systemic physiological dysfunctions including those in the gastro-intestinal tract^1,2^. Such dysfunctions manifest as autonomic imbalances in the gut and disrupt intestinal motility, mucosal secretions, and barrier permeability^3–5^. Recent studies have indicated differences in microbiome composition between adults with SCI or with multiple sclerosis vs those without CNS injuries^6–8^. This imbalance is exacerbated by frequent antibiotic use, physical inactivity, and psychological stress, which collectively disrupt the gut microbiome and promote the growth of inflammation-associated organisms^4,9^.

The gut microbiome influences distant organs and the CNS through crucial communication pathways such as the gut microbiome-CNS axes^10^. It does so via metabolites and direct interactions with the intestinal epithelia. Likewise, alterations in the microbiome composition can modulate the gut's immune system by disrupting the intestinal barrier leading to cytokine production and triggering adaptive immune response^11,12^. These cytokines can be either pro-inflammatory or anti-inflammatory and some may serve dual roles across various processes depending on the physiological context^13^. The link between inflammatory markers and the gut microbiome is not yet extensively studied but the available evidence suggests that interventions with probiotics and prebiotics can reduce inflammatory cytokines^14,15^, mitigate oxidative stress and improve nutritional status^16,17^.

Probiotics, comprising of diverse beneficial microbial species, have been shown to improve gut health^18^ and alleviate stress-induced symptoms^19^. Prebiotics, on the other hand, nourish and enhance healthy microflora^20^. Post-SCI administration of probiotics may normalize gut microbiota^4^, modulate the gut microbiome-CNS axis^10,21^ and improve health indices in this population^22^. Competitive athletes, including those who use wheelchairs, often adhere to well-balanced diets^23^. To address the gastrointestinal issues, athletes adopt a carbohydrate-rich, low-fiber diet, particularly before competitions, which could alter bowel motility and gut microbiome composition^24^. Notably, gastrointestinal illness was the third most reported ailment during the last three Paralympic Games following respiratory and skin illnesses^25^. Interventions with probiotics or prebiotics may offer benefits in terms of improving gut symptoms and overall gut health^18^.

Given these considerations, we conducted a cross-over randomized controlled pilot study involving elite athletes in wheelchair. This study aimed to explore the feasibility of the approach but also serve as an indication whether probiotics compared to prebiotics can improve inflammatory status and gut microbiome composition in elite athletes in wheelchair. Furthermore, this pilot trial is designed to inform the design of a larger and more comprehensive study.

## Materials and methods

The protocol and feasibility criteria for this trial have been previously published^26,27^. Recruitment started 21/03/2021 and last day of follow up was 29/10/2021. A brief summary is provided below for reference.

### Setting and study population

This study was a single-blinded, randomized, cross-over clinical trial conducted at the Sports Medicine of the Swiss Paraplegic Center, Nottwil, Switzerland. The study population comprised of elite athletes in wheelchair shortlisted for the Tokyo 2020 Paralympic games or actively participating in international competitions. Exclusion criteria included those with gastrointestinal disease diagnosis, pregnancy, or current antibiotic use. Written informed consent was obtained from all participants. The study was conducted according to the Declaration of Helsinki and Swiss law, approved by the Swiss Ethics Committee for Northwest/Central Switzerland (EKNZ, project ID: 2020-02337), and registered last at ClinicalTrials.gov (NCT04659408 09/12/2020).

### Intervention and randomization

Participants were randomized to a four-week regimen to either a 3 g sachet of commercially available, freeze-dried, multispecies probiotic preparation (Bactosan pro FOS,Mepha, Basel, Switzerland) or a prebiotic—5 g (one teaspoon) of oat bran (Naturaplan, Coop, Switzerland) (Supplemental Fig. 1). The probiotic contained *Bifidobacterium lactis* W51, *Bifidobacterium lactis* W52, *Enterococcus faecium* W54, *Lactobacillus acidophilus* W22, *Lactobacillus paracasei* W20, *Lactobacillus plantarum* W21, *Lactobacillus salivarius* W24, *Lactococcus lactis* W19. Athletes were instructed to maintain their usual diet and training routine during the entire study. Due to the differences between both supplementations, blinding was not possible. We performed 1:1 randomization with blocks of two and four executed by a Good Clinical Practice compliant data management system (secuTrial®, Interactive Systems, Berlin). Initially, seven athletes were randomized to the probiotic and seven to the prebiotic intervention. At baseline (T0), the following parameters were collected: age, sex, length, waist circumference, medical diagnosis and/or SCI characteristics, presence of diseases, type of sport, average number, and duration of training. After the first supplementation phase (4 weeks), a washout period (four weeks) and the second crossover supplementation phase (four weeks) followed. Blood, stool, Gastrointestinal Quality of Life Index (GIQLI) score, and clinical details were collected at four timepoints.

### Blood inflammatory markers measurement and analysis

Serum was processed and stored at the SwiSCI Biobank, Nottwil, Switzerland. Blood was collected in serum monovette with ethylenediaminetetraacetic acid (EDTA) (Sarstedt, Switzerland) and serum was isolated after centrifugation and stored at − 80 °C. A multi-plex analysis was performed to measure in serum (LEGENDplexTM bead-based immunoassays, Biolegend GmbH, Germany) a range of inflammatory and metabolic blood inflammatory markers (growth factors, cytokines, chemokines, and adipokines) at all-time points.

The multi-plex analysis was performed with Human Essential Immune Response Panel 13-plex (cat. 740929; including Interleukin (IL)-4, IL-2, interferon gamma-induced protein (IP)-10, IL-1β, tumor necrosis factor (TNF)-α, monocyte chemoattractant protein (MCP)-1, IL-17A, IL-6, IL-10, interferon (IFN)-γ, IL-12p70, IL-8 and free active transforming growth factor (TGF)-β1); Human Vascular Inflammation Panel 1- C-reactive protein (CRP) (cat. 740591); Human Growth Factor Panel 13-plex (cat. 740180; including Angiopoietin2, epidermal growth factor (EGF), erythropoietin (EPO), fibroblast growth factor (FGF)-basic, granulocyte-colony-stimulating factor (G-CSF), granulocyte-macrophage (GM)-CSF, hepatocyte growth factor (HGF), macrophage (M)-CSF, platelet-derived growth factor (PDGF)-AA, PDGF-BB, stem cell factor (SCF), TGFα and vascular endothelial growth factor (VEGF)) and Human Metabolic Panel 4-plex (cat. 740212; including adiponectin, adipsin, leptin and resistin), by flow cytometer (CytoFlex, Beckman Coulter, Switzerland) and data were analyzed with LEGENDplex™ Biolegend software.

### Gut microbiome measurement and analysis

Stool samples were collected using OMNIgene® GUT (DNA Genotek, Ottawa, Canada) tubes and stored at – 80 °C at SwiSCI Biobank, Nottwil, Switzerland. DNA was then extracted using QIAamp PowerFecal Pro DNA Kit (QIAGEN, Hilden, Germany) as per the manufacturer's protocol. Qubit dsDNA BR Assay Kit using Qubit 4.0 fluorimeter (Thermo Fisher Scientific) was used to measure DNA concentrations.

Full length 16S sequencing was then performed by targeting the hypervariable regions 1–9 (V1–V9) (~ 1500 bp fragments) using the MinION nanopore sequencer (Oxford Nanopore Technologies, Oxford, UK). The sequencing libraries were prepared via Nanopore Ligation Sequencing Kit—SQK109 (Oxford Nanopore, Oxford, UK). The bacterial DNA was amplified using a set of 16S universal primers: forward primer 27F (5ʹ-AGAGTTTGATCCTGGCTCAG-3ʹ) and reverse primer 1492R (5ʹ-CGGTTACCTTGTTACGACTT-3ʹ) and barcoded using the PCR Barcoding Expansion 1–96 (Oxford Nanopore Technologies, EXP-PBC096). The amplicons were tagged with 5ʹ-TTTCTGTTGGTGCTGATATTGC-3ʹ for forward primers and 5ʹ-ACTTGCCTGTCGCTCTATCTTC-3ʹ for reverse primers. Sequencing was conducted after the quality control and priming of the flowcell (Flow Cell Mk I, R9.4, FLO-MIN106D) with 50 fmol of DNA library for 12 h using MinION Mk1C device (Oxford Nanopore Technologies). Basecalling was performed with Guppy (version 6.3.7) agent integrated in the EPI2ME software (version 5.2.13, Oxford Nanopore technologies). Barcodes were trimmed and sequences were filtered to include only those with a q-score ≥ 9. We then ran only the pass reads with Kraken 2 (18) and used the Silva database for taxonomic classification.

### Data analysis

Results were analyzed using R (R Core Team, 2016, Vienna, Austria, version 4.2.2). Descriptive statistics were employed for the inflammation data by reporting mean and standard deviation (SD). Median and interquartile range difference, when applicable, is reported and specified. For measures below detection levels, the lowest detectable value from the assay were used. A hierarchical clustering using ward. D2 was run with the baseline inflammatory cytokine data and the groups were cross-checked with the values of the cytokines. Groups were then labelled based on profiles as high or low inflammatory status. Low inflammatory status samples were those with low values or undetectable in all measurements. Inflammation status is used when pertaining to the overall profile. A linear mixed-effect model (LMM) was used in R using the package lme4 for comparing the changes in inflammatory markers after use of probiotic vs prebiotic. The intervention (prebiotic coded as 0 and the probiotic as 1) in relation to the time of measure (before and after) were the fixed-effect (intervention*time) and the participants as random effects. The sequence of administration of the intervention was likewise controlled. Both models were compared by Analysis of Variance (ANOVA). Significant differences were noted when the P value was less than 0.05. The microbiome data were analyzed at the genus levels. The alpha diversity was measured using the observed richness, Chao1, Shannon, and Simpson indices. The alpha diversity was analyzed using LMM as described above. The microbiome data was then transformed by center-log ratio and the beta diversity was visualized using Principal Component Analysis (PCA). Beta diversity difference were tested using both by permutational multivariate analysis of variance (PERMANOVA) and permutational multivariate analysis of dispersion (PERMDISP). Differential abundances was tested using ANOVA-like differential expression (ALDEx2)^28^.

## Results

### Baseline characteristics

Fourteen participants participated in this pilot study (Table 1) and completed the trial. Outcomes related to recruitment, eligibility, retention, and feasibility criteria have been reported previously^27^. Demographic details are summarized in Table 1. Stool samples were collected from all athletes across four visits with one sample missing.

**Table 1:** The demographics of participants in the trial.

Characteristics	Study population (n = 14)
Personal and SCI characteristics	
Age, years, mean (SD)	33.9 (9.4)
Sex, n (%)	
Male	6 (42.9)
Female	8 (57.1)
Education	
High-school	1 (7.1)
Vocational training	8 (35.7)
University	8 (57.1)
Etiology of injury, n (%)	
Traumatic	6 (42.8)
Congenital	6 (42.8)
Multiple sclerosis	2 (14.3)
Injury level, n (%)	
Cervical	2 (14.3)
Thoracic	9 (64.3)
Not-defined	3 (21.4)
Sports	
Type of sport, n (%)	
Tennis/badminton	4 (28.6)
Hand bike	4 (28.6)
Wheelchair racing	3 (21.4)
Other	3 (21.4)
Weekly engagement in sport activities, mean (SD)	
Training per week, hours	8.1 (2.9)
Exercise hours per week, hours	13.8 (4.8)
Anthropometric data, mean (SD)	
BMI, kg/m^2^	21.9 (4.2)
Waist circumference, cm	81 (6.7)
Fat mass, kg	18 (7.8)
Fat free mass, kg	40.1 (7.6)
Medication/supplements use, n (%)	
Bladder muscle relaxants	3 (21.4)
Corticosteroids	2 (14.2)
Antidepressants	1 (7.1)
Vitamin D	7 (50.0)
Protein	7 (50.0)
Sports drinks	3 (21.4)
Omega-3	1 (7.1)

The mean waist circumference was 81 cm (6.7) (males: 82.3 [6.7]; females 80.0 [7.1]). The mean BMI was 21.9 kg/m^2^ (4.2) (males: 20.0 [1.7]; females: 23.5 [5.0]). Fat mass and fat-free mass were 18 kg (SD 7.8) and 40.1 kg (7.6), respectively. Supplement use was reported as follows: proteins (50%), vitamin D (50%), sports drinks (21.4%), and omega-3 fatty acids (7.1%). Additionally, the athletes used bladder muscle relaxants (21.4%), corticosteroid medications (14.2%) and antidepressants (7.1%).

### Baseline inflammatory status and gut microbiome

Baseline (T0) inflammatory markers are summarized in Table 2. The percentage of athletes in wheelchair with inflammatory markers below the detection level ranged between 7.1% and 57.1% with IL-1β being the most frequent below detection limit (Table 2). Predominantly, most of the athletes in wheelchair had low inflammation status at baseline, although higher baseline levels of inflammatory markers were found in 4 individuals (n = 2 SCI; n = 1 spina bifida; n = 1 arthrogrypose).

**Table 2:** Baseline measurements of serum inflammatory markers of participants included in the trial.

Inflammatory markers	Median (IQR)	% of athletes below detection limit	Assay detection limit
Essential immune response			
IL1β, pg/ml	27.7 (163.1)	57.1	27.7
IL2, pg/ml	15.1 (152.5)	14.3	3.0
IL4, pg/mL	135.2 (1395.9)	14.3	4.5
IL6, pg/ml	90.1 (1584.2)	28.5	20.0
IL8, pg/ml	11.6 (35.2)	50.0	9.0
IL10, pg/ml	18.3 (132.8)	7.1	3.5
IL12p70, pg/ml	44.2 (338.1)	35.7	9.2
IL17A, pg/ml	4.2 (43.2)	28.5	1.1
IFNγ, pg/ml	61.7 (342.9)	35.7	20.0
IP10, pg/ml	98.8 (90.6)	7.1	9.2
MCP1, pg/ml	173.2 (84.6)	0.0	0.5
TNFa, pg/ml	15.3 (209.5)	28.5	4.0
TGFβ1, pg/ml	163.3 (574.1)	35.7	65.2
CRP, mg/l	0.9 (1.6)	0.0	0.07
Growth factors			
Angiopoietin2, ng/ml	0.8 (0.4)	0.0	0.1
PDGF-AA, ng/ml	26.7 (10.1)	0.0	0.02
PDGF-BB, ng/ml	8.0 (5.0)	0.0	0.01
SCF, pg/ml	108.4 (280.5)	0.0	2.7
TGFα, pg/ml	31.7 (171.1)	7.1	4.8
VEGF, pg/ml	166.6 (169.6)	7.1	63.9
HGF, ng/ml	0.5 (0.5)	0.0	0.02
EGF, pg/ml	92.0 (74.7)	0.0	2.4
EPO, mU/ml	15.9 (28.0)	50.0	15.2
FGF-basic, pg/ml	56.9 (288.6)	28.6	2.8
G-CSF, pg/ml	84.5 (274.3)	14.3	24.4
GM-CSF, pg/ml	15.0 (113.7)	42.9	11.7
Metabolic factors			
Adipsin ng/ml	2085.0 (626.7)	0.0	5.9
Leptin ng/ml	5.9 (9.2)	21.3	4.0
Resistin ng/ml	1.3 (0.6)	0.0	0.5

Measured markers are both pro and anti-inflammatory.

**Figure 1:**
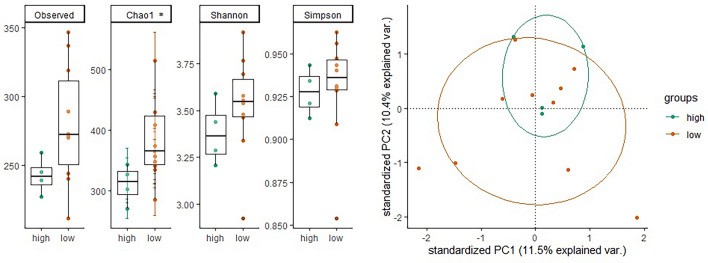
The association between the inflammation status and the gut microbiome of Swiss athletes in wheelchair. The alpha diversity (left) is presented with lower diversity among wheelchair athletes with low inflammation status vs high inflammation. Only Chao1 was with significant difference. The beta diversity (right) is presented and there is higher dispersion as measured via PERMDISP (p-value 0.063) in the low inflammation status vs high inflammation.

We stratified the participants into two groups based on low (n = 10) and high (n = 4) baseline inflammation statuses and compared their gut microbiome. Alpha diversity was significantly higher in the low inflammation group across all four indices (Fig. 1). Beta diversity analysis revealed a higher dispersion in the low inflammation group compared to high inflammation group. No significant taxa difference was found in differential abundance analysis.

### Inflammation status after probiotic and prebiotic administration

The changes in the inflammatory marker are summarized in Table 3. In 25/30 (83%) of inflammatory markers, the use of probiotic had higher decrease in values compared to after prebiotic use. In five markers (angiopoietin 2, GMCSF, SCF, TGFa, and adipsin), the use of probiotic had higher increase values compared to prebiotic use. No significant differences were observed. The GIQLI scores likewise showed higher values in scores after use of probiotic compared to prebiotic use. No significant difference was observed. We controlled for sequence of administration of intervention and found no significant difference in the models. Plots of the inflammatory markers across the time points for intervention period are in Supplemental Figs. 2–4.

**Table 3:** Summary of the changes of the serum inflammatory markers comparing the use of probiotic vs prebiotic.

Biomarker	Coefficient	SE
IL4	− 66.78	107.69
IL2	− 13.74	18.97
IP10	− 7.08	15.46
IL1b	− 11.58	10.43
TNFa	− 23.44	24.10
MCP1	− 6.66	17.74
IL17a	− 4.22	5.24
IL6	− 499.68	437.86
IL10	− 14.72	17.61
IFNg	− 89.02	116.14
IL12p70	− 62.43	72.70
IL8	− 14.31	26.65
TGFb1	− 46.69	54.50
Angiopoietin2	0.05	0.12
EGF	− 2.92	11.64
EPO	− 5.36	7.10
FGF	− 17.45	184.06
GCSF	− 4.98	71.50
GMCSF	10.04	23.27
HGF	− 0.09	0.17
MCSF	− 2.16	23.77
PDGFAA	− 3.33	3.73
PDGFBB	− 0.12	0.70
SCF	19.20	38.63
TGFa	1.85	34.13
VEGF	− 5.23	20.58
Adipsin	10.80	295.61
Leptin	− 1.13	0.89
Resistin	− 0.19	0.18
CRP	− 0.03	0.48
GIGLI	4.93	5.78

Measured markers are both pro and anti-inflammatory. The coefficient denotes the magnitude of change in the values of the markers after use of probiotic vs prebiotic. Negative values indicate greater decrease in inflammatory measure after use of probiotic compared to prebiotic while a positive value indicate a greater increase. The SE is the standard error of the coefficient.

### Microbiome post-intervention

After probiotic use had significantly higher increased in observed taxa compared to after prebiotic use. Likewise, the Chao1, Shannon and Simpson had higher increase in diversity after use of probiotic vs use of prebiotic but were not significantly different (Table 4). Beta diversity did not significantly differ following either use of probiotic or prebiotic (Supplemental Fig. 4). No significant differential bacterial taxa were identified after use of either intervention. Interestingly, the genus *Enterococcus* had higher relative abundance (effect size 0.71) after probiotic use compared to after prebiotic use. One of the components of the probiotic was *Enterococcus faecium* W54. Comparing the differential abundance between before and after use of the probiotic, *Enterococcus* (effect size 0.59) had higher relative abundance after use and groups of *Ruminococcaceae* (effect size 0.39–0.49) had lower relative abundance (Supplemental Fig. 6).

**Table 4:** Summary of the changes of the alpha diversity indices comparing the use of probiotic use vs the prebiotic.

Index	Coeff	SE
Observed	32.70*	14.65
Chao1	50.40	25.56
Shannon	0.05	0.10
Simpson	0.01	0.85

The coefficient denotes the magnitude of change in the diversity after use of probiotic vs prebiotic. Negative values indicate higher decrease in diversity after use of probiotic compared to prebiotic while a positive value indicate a higher increase. The SE is the standard error of the coefficient.

*p value < 0.05.

## Discussion

This pilot trial primarily focused on elite athletes in wheelchair, most of whom exhibited low inflammatory status. The study revealed an association between low inflammation status and higher gut microbiome diversity. No significant changes were found in all the inflammatory markers but the use of probiotic had greater decrease in inflammatory markers compared to use of prebiotic. Inflammatory markers measured included both pro- and anti-inflammatory markers. No significant difference was observed when the sequence of the administration was controlled for in the LMM indicating that the washout period was sufficient. The gut microbiome diversity had greater increase after use of probiotic vs use of prebiotic but the community composition remained stable after supplementation. Differential abundance analysis of the gut microbiomes likewise shows that one of the components of the probiotic, *Enterococcus faecium W54*, could be influencing the abundance of the genus *Enterococcus* after probiotic use.

Athletes typically maintain a low inflammatory status and acute inflammation post-physical activities but they have a balance pro- and anti-inflammatory cytokines in the long-term^29–31^. Variability in inflammation levels is attributable to differences in training intensity, the type of sport practiced, and the inflammatory markers used in the studies^32–36^. Individuals with SCI and those with multiple sclerosis, the population under study in this pilot trial, generally exhibit low grade chronic inflammation status characterized by elevated levels of circulating cytokines^37,38^. The majority of our participants demonstrated low concentrations of circulating cytokines. This suggests that physical activity may aid in lowering systemic inflammation in individuals with SCI or multiple sclerosis. This aligns with existing literature suggesting that long-term physical activity may reduce systemic inflammation possibly due to do decrease in the amount of adipose tissue^39–41^.

The use of probiotics had higher decrease in inflammatory markers compared to the use of prebiotics. The prebiotics used in the study was oat bran at 5 g, which is a low dose, and we did not expect significant physiological effect. The probiotics used are lactic acid bacteria that could decrease intestinal pH, thereby inhibiting the growth of pathogenic bacteria. Current probiotics are hypothesized to modify the gut microbiome, competitively adhere to the gut mucosa, modulate the immune system, strengthen the epithelial barrier with minimal tissue damage but these mechanisms are not fully explained^42^. The effect is transient and there is a need for high amounts of the live probiotics to exert health benefits. The genus *Enterococcus* was observed to have higher relative abundance after use of the probiotic when compared to after prebiotic use in the differential abundance analysis. Likewise, comparing before and after probiotic use, the genus *Enterococcus* had higher relative abundance after probiotic use while the genera of the other components did not have high effect sizes. This indicates that the *Enterococcus faecium W35* might be able to colonize the gut successfully or influence the growth of other *Enterococcus* in the gut. It is important to note though that multiple species of *Enterococcus* are common gut bacteria in adults. Our analysis did not target species or strain level classification and thus we could not fully elucidate whether *Enterococcus faecium W35* is the species or strain with high relative abundance. Moreover, the use of probiotics had lower relative abundance of groups of *Ruminococcaceae*. Certain members of the family are pathobionts and are able to initiate inflammatory responses in the gut^43,44^. The observed results are not statistically significant but the general trends after use of probiotics indicate towards lower inflammatory markers and therefore validation in bigger trials are needed.

An association between inflammation status and gut microbiome diversity indices was also observed in this trial. The low inflammation status group at baseline corresponded with higher gut microbiome alpha diversity and had difference in dispersion compared to the high inflammation status group. However, no significant difference in global composition was observed. Likewise, the use of probiotic led to higher diversity compared to prebiotic use but no difference in the composition. This indicates that the compositional changes in the microbiome due to inflammation could be random and the microbiome composition is stable. The exposure to the interventions were only four weeks and may need more time before any significant changes can be observed. It is important to take note as well is that the analysis conducted in the trial was on the microbiome taxonomy and may not reflect the functional changes in the gut microbiome community. Evidence in literature also shows that effects of probiotics are transient and may not have been captured in the analysis.

We have observed that the use of probiotic had greater increase in GIQLI scores compared to the use of prebiotic. The higher GIQLI indicates a better gut health condition. This in line with the results in the inflammatory markers and gut microbiome. The use of probiotic had a greater rate of decrease in inflammatory markers and a greater rate of increase in gut microbiome diversity compared to the use of prebiotic though the changes are not statistically significant. Moreover, when we compared the models when we controlled for the sequence of administration for the inflammatory markers, gut microbiome alpha diversity and GIQLI scores, we found no significant differences. This indicates that the washout period was sufficient and thus the carry-over effect from one intervention to the other is minimal.

As a pilot trial, significant changes were not expected due to the small sample size and thus we focused on the general direction of the effect sizes of the interventions tested. The prebiotic used in the study was oat bran at 5 g and this amount may not have significant physiological effect in the gut^26,45^. In effect, the prebiotic treatment is acting as a control. Thus, the comparison between use of probiotic vs prebiotic could be misinterpreted as if the probiotic is better than the prebiotic. A higher amount of prebiotic, ranging from 40 g of oats or higher^45^, might be needed to detect an effect. Likewise, probiotics contains varying strains of organisms and could exert different effects in the gut. One of the questions in probiotics is whether the organisms have the ability to engraft and multiply in the gut before they exert an effect or whether their presence alone has an effect. In multistrain and multispecies probiotics, the interaction of the organisms could likewise exert an effect.

The majority of participants had low inflammation status at baseline and the changes in the inflammatory markers post-intervention were minimal. Athletes with high inflammation status have higher difference after intervention. Choosing a population with higher baseline inflammation status could better show the effect of probiotics or of prebiotics in reducing inflammation. In addition, the included athletes in wheelchair had multiple cause of their wheelchair-dependency which may influence the inflammatory and gut microbiome status in the study. The trial was also conducted during the training and competition phases. The beginning of the trial was during training and after crossover many of the athletes were in the competition phase. The changes in physical activity, diet, medication and supplement intake during this time may have influenced the inflammation and gut microbiome status of the athletes in wheelchair.

The future trial would benefit from increasing the sample size and focusing on populations with higher inflammation status particularly non-athletes when using a probiotic or prebiotic as an intervention. Longer intervention exposure is recommended especially when gut microbiome diversity indices are used. Identifying individual taxa differential abundance rather than relying on the microbiome diversity indices should be considered. Since there are multiple differential abundance methods that are available, use of multiple methods and use of consensus of the results is advisable^46^.

## Conclusions

Most of the elite Swiss athletes in wheelchair in this trial had low inflammatory status, which corresponded with higher gut microbiome diversity. The trial is a pilot, and while the probiotics used show potential, validation in a larger trial is needed. The direction of the effect sizes, though not significant, indicates a greater decrease in inflammatory markers in the probiotic group compared to the prebiotic group. For future trials, the focus should be on having sufficient sample sizes, populations with higher inflammation levels, longer intervention periods, and use of differential abundance analysis to detect changes after intervention.

### Supplementary Information

Supplementary Figures.

## Data Availability

Data is provided within the manuscript and supplementary information files. Data can be requested to the primary author upon reasonable request.
