# Supplementary material for: Gut microbiome and inflammation among athletes in wheelchair in a crossover randomized pilot trial of probiotic and prebiotic interventions
Source: Sci Rep. 2024 Jun 4;14:12838. doi: 10.1038/s41598-024-63163-z (PMC11150429; doi:10.1038/s41598-024-63163-z)
Supplement: Supplementary file 1 — Supplementary Figures. [file 41598_2024_63163_MOESM1_ESM.docx]

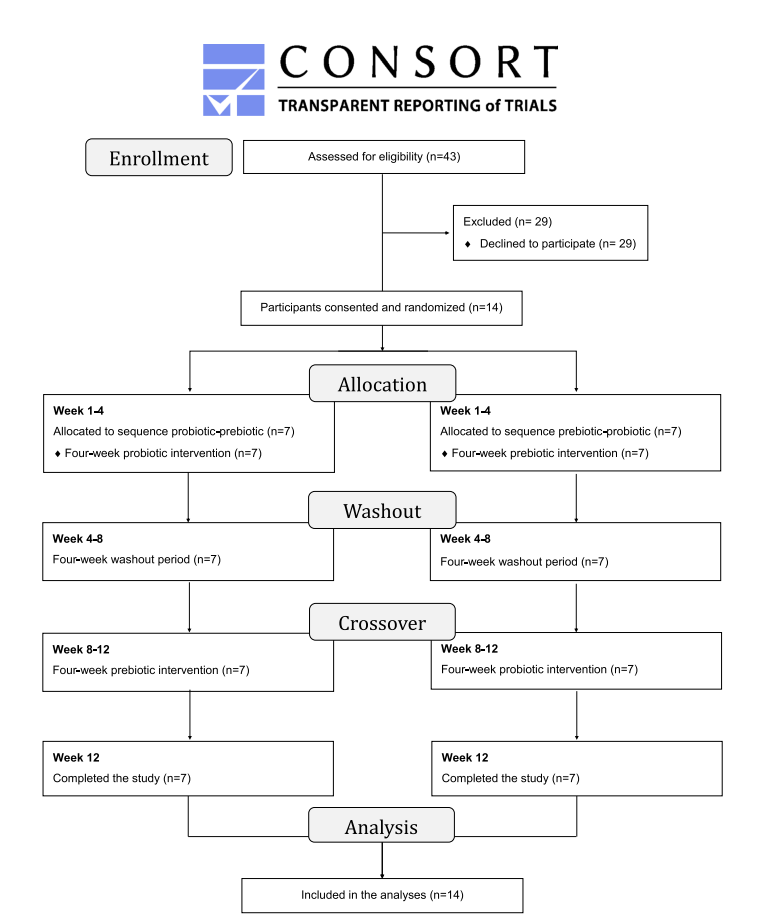


Supplemental Figure 1. The study workflow of the trial [1]. Image taken with permission.

1. Hertig-Godeschalk A, Glisic M, Ruettimann B, Valido E, Capossela S, Stoyanov J, Flueck JL. The feasibility of a randomized controlled crossover trial to assess the effect of probiotic and prebiotic supplementation on the health of elite wheelchair athletes. Pilot Feasibility Stud. 2023;9(1):99.


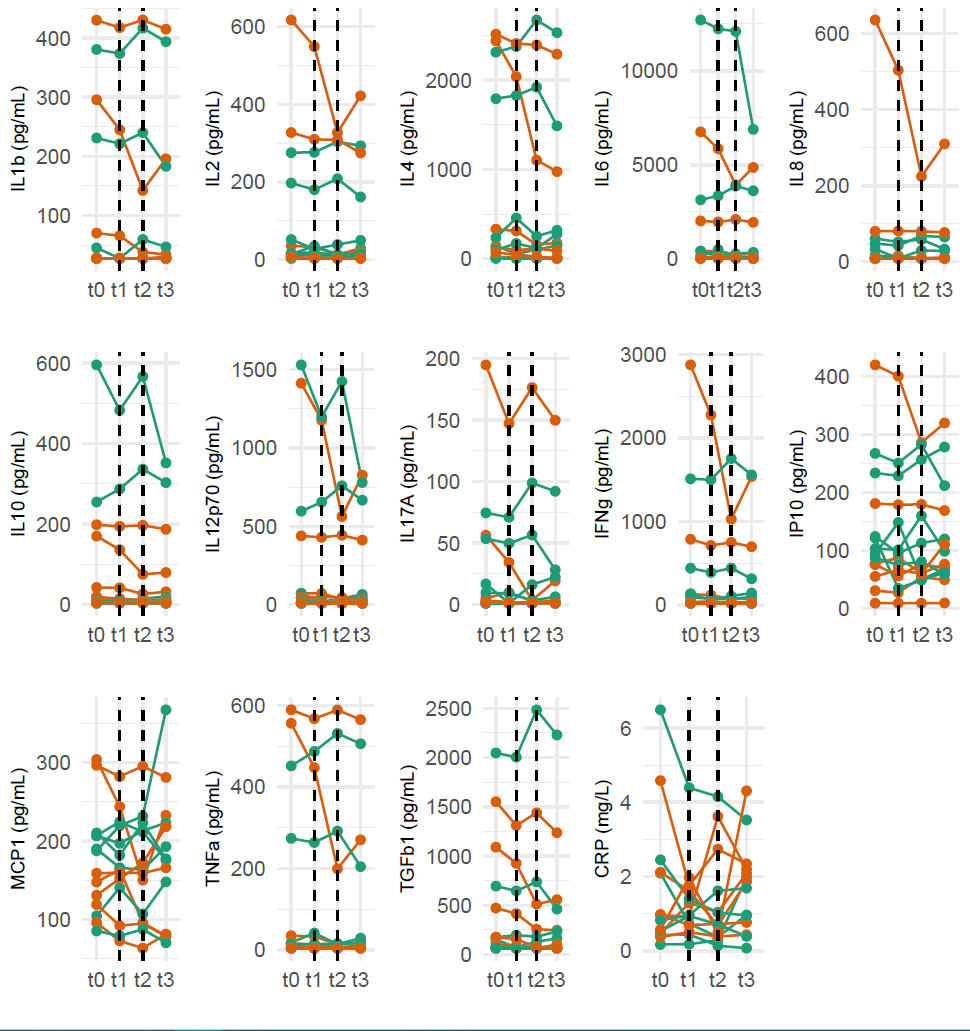

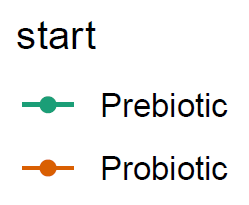


Supplemental Figure 2. The concentration plots of the inflammatory markers measured in all time points in the trial from all participants. t0 denotes the baseline timepoint. t1 and t2, represented with vertical dashlines, denotes the washout period. The color specify the starting intervention either with the prebiotic (green) or probiotic (orange).


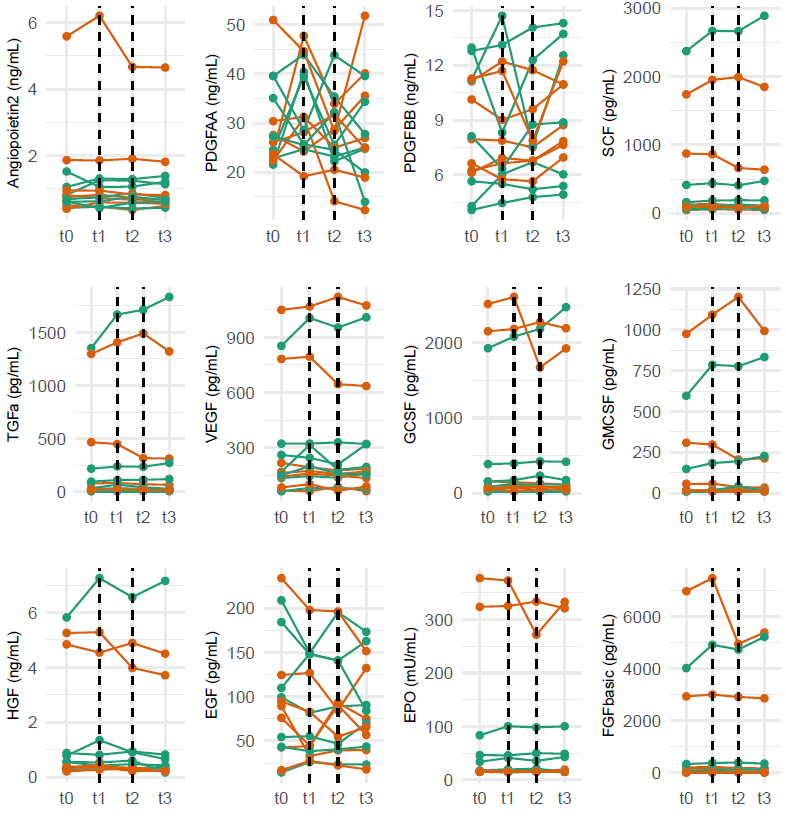

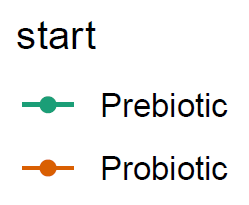


Supplemental Figure 3. The concentration plots of the inflammatory growth factors measured in all time points in the trial from all participants. t0 denotes the baseline timepoint. t1 and t2, represented with vertical dashlines, denotes the washout period. The color specify the starting intervention either with the prebiotic (green) or probiotic (orange).


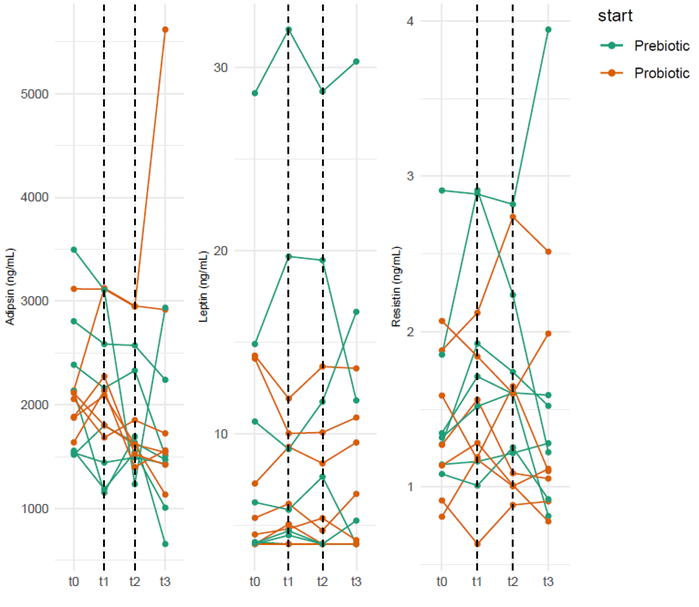


Supplemental Figure 4. The concentration plots of the metabolic inflammatory markers measured in all time points in the trial from all participants. t0 denotes the baseline timepoint. t1 and t2, represented with vertical dashlines, denotes the washout period. The color specify the starting intervention either with the prebiotic (green) or probiotic (orange).


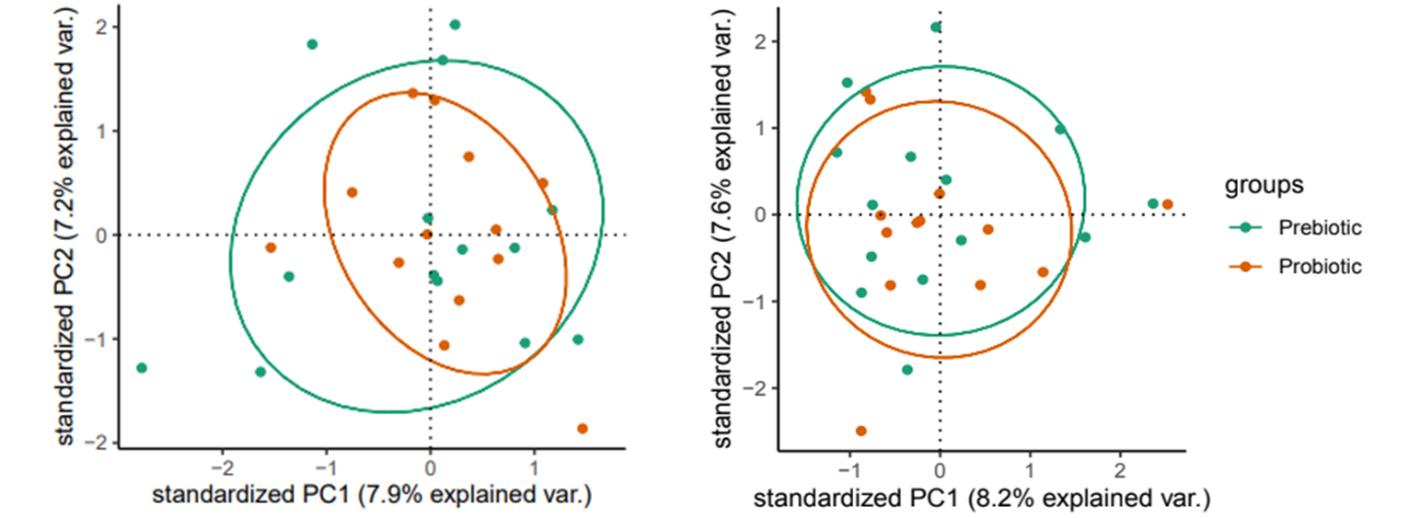


Supplemental Figure 5. The principal component analysis plots for the before the supplementation (left) and after (right). There was no significant difference in both the location and dispersion of the beta diversity of the groups.


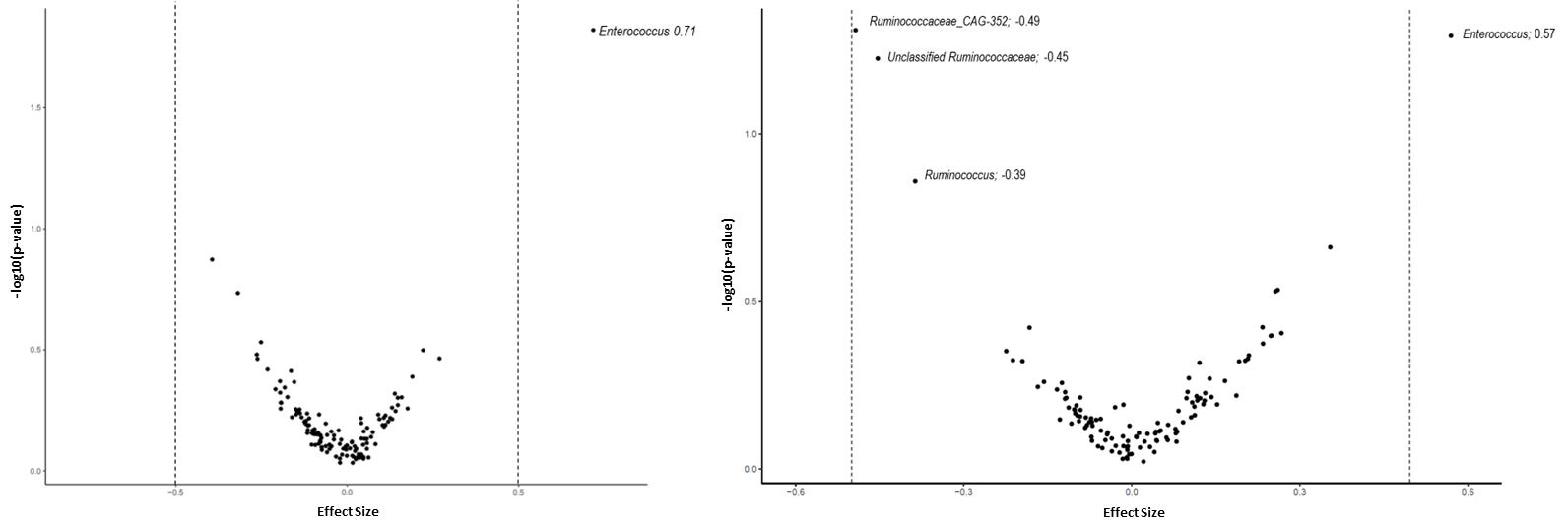


Supplemental Figure 6. This is the volcano plots of the standardized effect sizes from the ANOVA-like differential expression 2 (ALDEx2) differential abundance analysis against the -log10 (P value). The plot on the left compares between after probiotic (positive side) and after prebiotic use (negative side). The plot on the right compares between before (negative side) and after (positive side) probiotic use. In both plots, each group had 26 samples. The higher the magnitude of the effect size on the positive side denotes higher relative abundance of the taxa in the group or more depleted in relative abundance compared to the opposite group. For example, using the plot on the left, *Enterococcus* has higher relative abundance in after probiotic use compared to after prebiotic use. The opposite is true in which *Enterococcus* has lower relative abundance in after prebiotic use compared to after probiotic use. No significant differential taxa were found after P value was adjusted with Bonferroni-Hochberg correction with cut-off at 0.05.
